# Effects of Meditation on Structural Changes of the Brain in Patients With Mild Cognitive Impairment or Alzheimer’s Disease Dementia

**DOI:** 10.3389/fnhum.2021.728993

**Published:** 2021-11-12

**Authors:** Madhukar Dwivedi, Neha Dubey, Aditya Jain Pansari, Raju Surampudi Bapi, Meghoranjani Das, Maushumi Guha, Rahul Banerjee, Gobinda Pramanick, Jayanti Basu, Amitabha Ghosh

**Affiliations:** ^1^Cognitive Science Lab, International Institute of Information Technology, Hyderabad, India; ^2^Department of Neurology, Apollo Gleneagles Hospital, Kolkata, India; ^3^Department of Applied Psychology, University of Calcutta, Kolkata, India; ^4^Department of Philosophy, Jadavpur University, Kolkata, India; ^5^Crystallography and Molecular Biology Division, Saha Institute of Nuclear Physics, Kolkata, India; ^6^Department of Radiology, Apollo Gleneagles Hospital, Kolkata, India

**Keywords:** meditation, mild cognitive impairment, Alzheimer’s disease, cortical thickness, gray matter volume, imaging, neuropsychology

## Abstract

Previous cross-sectional studies reported positive effects of meditation on the brain areas related to attention and executive function in the healthy elderly population. Effects of long-term regular meditation in persons with mild cognitive impairment (MCI) and Alzheimer’s disease dementia (AD) have rarely been studied. In this study, we explored changes in cortical thickness and gray matter volume in meditation-naïve persons with MCI or mild AD after long-term meditation intervention. MCI or mild AD patients underwent detailed clinical and neuropsychological assessment and were assigned into meditation or non-meditation groups. High resolution T1-weighted magnetic resonance images (MRI) were acquired at baseline and after 6 months. Longitudinal symmetrized percentage changes (SPC) in cortical thickness and gray matter volume were estimated. Left caudal middle frontal, left rostral middle frontal, left superior parietal, right lateral orbitofrontal, and right superior frontal cortices showed changes in both cortical thickness and gray matter volume; the left paracentral cortex showed changes in cortical thickness; the left lateral occipital, left superior frontal, left banks of the superior temporal sulcus (bankssts), and left medial orbitofrontal cortices showed changes in gray matter volume. All these areas exhibited significantly higher SPC values in meditators as compared to non-meditators. Conversely, the left lateral occipital, and right posterior cingulate cortices showed significantly lower SPC values for cortical thickness in the meditators. In hippocampal subfields analysis, we observed significantly higher SPC in gray matter volume of the left CA1, molecular layer HP, and CA3 with a trend for increased gray matter volume in most other areas. No significant changes were found for the hippocampal subfields in the right hemisphere. Analysis of the subcortical structures revealed significantly increased volume in the right thalamus in the meditation group. The results of the study point out that long-term meditation practice in persons with MCI or mild AD leads to salutary changes in cortical thickness and gray matter volumes. Most of these changes were observed in the brain areas related to executive control and memory that are prominently at risk in neurodegenerative diseases.

## Introduction

Alzheimer’s disease is a clinico-pathological continuum caused by abnormal deposition of misfolded beta-amyloid and tau proteins with progressive breakdown along the core neurocognitive networks in the brain. Clinical progression comes much later and proceeds from a preclinical state, through mild cognitive impairment to dementia (Petersen et al., 2001; Albert et al., 2011; Dipasquale and Cercignani, 2016; Jack et al., 2018). Annually, around 10–15% of patients with MCI could convert to Alzheimer’s disease dementia (hitherto referred as “AD”), Petersen et al. (2001) and over half of all converters are likely to do so within 5 years (Gauthier et al., 2006). Despite significant conceptual advances, the absence of effective disease-modifying drugs is frustrating and is pushing researchers to find alternative options to delay disease progression.

Of late, studies on meditation have consistently demonstrated structural and functional changes in the brain, including in gray matter volume, cortical thickness, and white matter tracts (Fox et al., 2014), regional cerebral blood flow (Newberg et al., 2010), and functional activity (Fox et al., 2016). During common meditation techniques, the meditator actively attends to breath, sensations, mantras, sounds or objects in what has been described as “focused attention” tasks, or to the rise and fall of thoughts and sensations in “open monitoring” tasks (Lutz et al., 2008). The default component is passive mind wandering, from where the meditator has to repeatedly turn the attention back to the task at hand. Other techniques such as loving-kindness meditation and meditation on peace, where faces, thoughts, or scenes are visualized, respectively, could be considered variants of “focused attention” meditation.

Hasenkamp and Barsalou (2012) demonstrated the effects of meditation on the core neurocognitive networks in healthy meditators. In healthy subjects, the activities of the default mode network (DMN) and the central executive network (CEN) are anti-correlated, activation of one being coupled with the deactivation of the other, for example, when a healthy person switches from self-referential thinking or mind wandering to a goal-directed focused task, or vice versa. The salience network (SN) recognizes the immediate salience of the task at hand and executes this switch (Seeley et al., 2007; Sridharan et al., 2008). Hasenkamp and Barsalou (2012) demonstrated that the same coordination between the three networks takes place during focused meditation. As a meditator moves from mind wandering to awareness of mind-wandering to switching back to focused breathing, there is corresponding activation of the DMN, SN, and CEN, respectively, until the cycle repeats. Indeed, meditation can be considered an exercise in cognitive control that has to pass through repeated phases of anti-correlated activation and deactivation of reciprocal neurocognitive networks.

A meta-analysis of studies in healthy meditators (Fox et al., 2014) showed that the most dependable morphometric differences in favor of meditation were found in the left rostrolateral prefrontal cortex (BA 10), anterior and mid-cingulate cortex, anterior insula, primary/secondary somatomotor cortices, left temporal gyrus and hippocampus. The meta-analysis, however, largely depended on studies on young to middle-aged meditators and did not include studies on patients with cognitive impairment. Reports on brain changes in elderly meditators are sparse (Luders et al., 2015; Chételat et al., 2017) and longitudinal studies in this cohort are lacking.

Few researchers have studied the effect of meditation (or mindfulness) on structural or functional changes in the brain in patients with MCI or AD (Newberg et al., 2010; Wells et al., 2013a,b; Yang et al., 2016; Fam et al., 2020; Yu et al., 2021). Morphometric studies are even rarer (Wells et al., 2013b; Yang et al., 2016; Yu et al., 2021) and none included patients with mild AD. In the structural MRI component of their studies, both Wells et al. (2013b) and Yang et al. (2016) assessed gray matter volume but not cortical thickness, whereas Yang et al. (2016) especially focused on the dorsal anterior cingulate cortex and bilateral hippocampi. Further, most of the studies so far have used various mind-body exercises rather than meditation alone. The Mindfulness-Based Stress Reduction (MBSR) protocol (Kabat-Zinn, 1990) followed by Wells et al. (2013b) includes body movements as one of the components. Yang et al. (2016) used Kirtan Kriya protocol that includes chanting of a mantra and finger movements during meditation. Movements also formed an important part of the protocol followed by Yu et al. (2021). All of these make it difficult to interpret the contribution of each component of the meditation protocol to the eventual structural or functional alterations in the brain. Moreover, Yu et al. (2021) used a low-intensity meditation protocol whereby patients were required to come weekly for the first 3 months and then monthly for the next 6 months. Follow-up assessments also varied between studies from 8 weeks to 9 months (Wells et al., 2013b; Yang et al., 2016; Yu et al., 2021).

Several unexplored or underexplored avenues therefore remain. For example, what would be morphometric brain changes following daily meditation if performed over a long term in both MCI and AD subjects? What would such changes be if only a silent, sitting meditation protocol is followed, without involving movements or vocalization in the process? Does repeated top-down modulation of behavior that is inherent in meditation practice bolster the regions of the brain that relate to executive control?

In this study, we try to address some of these issues as we investigate the impact of long-term daily silent, sitting meditation on brain gray matter volume and cortical thickness in MCI and mild AD patients. Given the limited data available in the literature on this topic, we plan to approach our query using whole-brain analysis for morphometric changes including the hippocampal subfields and other subcortical structures rather than focusing on *a priori* regions of interest. We hypothesize that long-term meditation in patients with MCI and mild AD will benefit brain areas that underlie attention and executive control. We also hypothesize that long-term meditation in these patients will not benefit the more severely affected posterior areas of the brain such as the posterior cingulate cortex (PCC).

## Materials and Methods

### Participants

In this hospital-based longitudinal study, patients who met the clinical criteria of either amnestic MCI (Winblad et al., 2004; Albert et al., 2011) or probable AD (Jack et al., 2011) were recruited between November 2018 and March 2020, based on the following criteria: age between 45 and 70 years; formal education up to at least 8th grade; no psychotherapy for at least 2 months before recruitment; medication prescribed for dementia or MCI should have been at a constant dose of at least 4 weeks before recruitment. Patients also needed to have a global Clinical Dementia Rating (CDR) score between 0.5 and 1 (Morris, 1993) and a Fazekas score of between 0 and 2 on their screening MRI scans (Fazekas et al., 1987). Subjects were excluded from the study if they had been practicing any form of meditation or mindfulness procedures, had unstable medical conditions, active or chronic major organ disease, strokes or traumatic brain injury or seizures within 12 months or with residual disability, history of multiple strokes or transient ischemic attacks (TIAs), obstructive sleep apnea, psychiatric disorders other than mild to moderate anxiety or depression, had been on newly prescribed psychotropic drugs or underwent dose change within the last 4 weeks. All participants were required to have a caregiver accompanying them.

Detailed information of the study was provided to each patient and written informed consent was obtained. The Institutional Ethics Committee of Apollo Gleneagles Hospital, Kolkata, India cleared the study.

Forty-eight eligible patients consented to participate in the study. Recruited subjects were assigned into a meditation group and control groups by a convenience sampling method. Control groups in turn were subdivided into an active control (non-meditation-focused task) group and a treatment-as-usual (TAU) group. Treatment as usual was kept constant for all groups and included anti-dementia medicines where indicated, avoidance of drugs that could exacerbate cognitive impairment, correction of reversible risk factors, and advice on lifestyle measures.

All recruited participants underwent a detailed assessment including clinical history, physical examination, and neuropsychological assessments by a cognitive neurologist and a clinical neuropsychologist. The neuropsychological tests were selected on the basis of the commonly reported changes in previous meditation studies and included the Montreal Cognitive Assessment (MOCA) (Nasreddine et al., 2005), CDR sum of boxes score (CDR-SB) (Morris, 1993; O’Bryant et al., 2008), digit span forward and backward, Trail Making Test (TMT)-black and white A and B (Kim et al., 2014), and the CERAD word list memory test (Morris et al., 1989). All patients underwent brain imaging. The researchers involved in MRI image acquisition (GP) and image analyses (MD, AJP, RSB) were blinded to the assigned groups as well as to the clinical and neuropsychological findings.

### Intervention Protocols

Participants in the meditation group completed training sessions over 2 weeks, under the direct guidance of a trainer. Most of the meditative procedures were culled from acclaimed classical sources such as the Patanjali Yoga Sutras (Vivekananda, 2001) and the Path of Purification (Buddhaghosa and Nanamoli, 2003; Banerjee and Chatterjee, 2018). Out of several procedures, a subset of four meditation procedures was selected, namely, full-body relaxation technique, meditation on peace, witnessing the breath, and witnessing of thoughts.

After a detailed explanation of the process and any required clarification, the trainer conducted a “guided meditation” wherein step-by-step instruction was provided to the patient to induce the meditative state. Each meditation protocol was treated as an independent unit and generally, one protocol was taught to the patient per class on a one-to-one basis. After four sessions over a 2-week period, a handout was provided to the patient describing the procedures in detail and a CD recording of the guided meditation provided for practice at home. Each session of guided meditation lasts about 30 min (1 min to settle down, 7 min per technique including around 10 s of transition time between each technique and 1 min to come out of meditation), which the patient was required to practice daily for the next 6 months.

• Full Body Relaxation Technique

Although this technique may not be considered meditation proper, it nevertheless precedes every meditation session. The meditator systematically surveys the entire body beginning with the feet and traveling to the head, consciously relaxing all muscular and nervous tension.

• Meditation on Peace

There are many variants to the meditation on peace. The procedure, which we have adapted for this study, has been simplified to enable all to perform. In this, the subject is encouraged to visualize a scene or suitably personalize a moment from the meditator’s past, which is experienced as being calm and tranquil and can evoke a feeling of peace. The meditator is encouraged to self-assert this feeling of peace during this visualization.

• Witnessing the Breath

In this, the subject is trained to concentrate on the natural and spontaneous flow of breath, without actively engaging in the process of inhalation or exhalation. The meditator gradually focuses the attention from chest movements to the nose to the nostrils and finally to a small point just below the nostrils where the out-breath brushes the upper lip on its way out. After anchoring attention to the breath, the meditator directs the attention to the various characteristics of the breath, including the in-breath and out-breath, their differences in temperature, and to changes in the length and rhythm of the breath. When extraneous thoughts arise, the meditator, on acknowledging the same, does not follow the thought but gently redirects the attention back to the breath.

• Witnessing of Thoughts

The subject is trained to observe the flow of thoughts in the attitude of a witness. This attitude implies an even-minded, detached observation of the flow of thoughts without regard to their specific content. The meditator is advised not to attempt to block the thoughts when they arise, nor to cling on to them but to simply observe how they appear and disappear spontaneously, and thereby acknowledge their transience. There must be clear apprehension of the thoughts and the meditator should ensure that she does not become drowsy or fall asleep.

Patients in the non-meditation-focused task group were given coloring books to work on for around 30 min daily for the next 6 months. When one set of coloring books was completed, another was provided to the patient. Patients were encouraged to choose their own colors and be imaginative.

Participants in both the meditation as well as the non-meditation focused task groups were provided with logbooks and encouraged to document their time logs. Regular follow–up phone calls were made to participants to check for compliance and to discuss issues related to performance, if applicable. Participants were also encouraged to contact the research team for any queries.

### MR Image Acquisition

High-resolution T1-weighted (T1-w) structural MRI scans were acquired using a 3T Philips Ingenia MRI scanner with a standard multi-channel head coil. Foam pads were used to minimize head movements and facilitate comfortable scan procedures. For morphometric analysis, three-dimensional T1-weighted Magnetization Prepared Rapid Gradient Echo (MPRAGE) pulse sequencing was used. The whole-brain 3D T1-weighted sequences were acquired with the following parameters: sagittal, the matrix size = 236 × 236, number of slices in each volume = 200, 1 × 1 mm^2^ in-plane resolution, slice thickness = 1.1 mm, Repetition Time (TR) = 6.8 ms, Echo time (TE) = 3.2 ms, flip angle = 8°. The images were reconstructed and manually checked for major artifacts (e.g., motion, ringing, warp around) before further preprocessing.

### Follow-up Assessment

By the end of 6 months of practice, 15 out of the 48 recruited patients (31%) had dropped out from the study, most of them doing so within the first 3 months. Another patient who developed early Parkinsonian symptoms was excluded from the rest of the study. Given the variability in visit timings because of restrictions during the Covid-19 pandemic, the second assessment took place at a median of 6.7 months (Q1, Q3: 6.2, 7.5 months, respectively). Out of the 32 patients who completed their second neuropsychological evaluation, 22 participants (11 in the meditation group, five in non-meditation focused task group, six in the TAU group) could complete their second MRI scanning, the rest of the participants being reluctant to undergo MRI scanning during the pandemic. Given the small number of imaged patients in the focused task group and the TAU group, we combined them into one “non-meditation group” for the analyses of imaging data.

### Image Processing

All high-resolution T1-w structural images were processed using publicly available Freesurfer software. Images were automatically processed with the processing pipeline related to the *longitudinal stream* in Freesurfer (Reuter et al., 2012). All scans from two time points (at time point 1 – the base scan and at time point 2 – the repeat scan) were first pre-processed independently with the *cross-sectional stream*. We have used the *Recon-all* stream for volumetric segmentation, which includes motion correction, skull stripping, intensity normalization, automated Talairach transformation, gray/white matter tessellation, and topology correction (Fischl et al., 2001; Ségonne et al., 2007). A base template was created for each participant from both the scans (at two-time points), which helps in the initial segmentation and surface reconstruction for structural data. For unbiased analysis in the longitudinal stream, measurements at the two-time points were registered to the base template. All segmented and surface reconstructed data were manually checked for segmentation accuracy for each time point. This preprocessing pipeline is widely used among researchers and provides high reliability and statistical power for structural data (Reuter et al., 2012). Freesurfer uses Desikan–Killiany Atlas (Desikan et al., 2006) to segment the cortex into 31 distinct cortical regions in each hemisphere based on the sulcal and gyral anatomy of the brain.

### Statistical Analysis

Statistical analysis was designed to take into account the variable time lags between visits when interpreting the structural brain changes and neuropsychological test scores. This was done by calculating the symmetrized percentage change (SPC) values for each test. SPC is defined as the rate of change with respect to the average values/thickness/volume [i.e., 100 × (annualized rate between time point 1 and time point 2)/average] (Reuter et al., 2012; Pegueroles et al., 2017). SPC can therefore be used to compare longitudinal differences between subjects even with variable inter time point intervals.


$$S\,y\,m\,m\,e\,t\,r\,i\,z\,e\,d\,p\,e\,r\,c\,e\,n\,t\,a\,g\,e\,c\,h\,a\,n\,g\,e=100*\frac{\left(t\,p\,1-t\,p\,0\right)}{a\,v\,e\,r\,a\,g\,e\,\left(t\,p\,0,t\,p\,1\right)*t\,i\,m\,e\,\_\,d\,i\,f\,f\,e\,r\,e\,n\,c\,e\,\left(t\,p\,0,t\,p\,1\right)}$$

Where, tp0 → measure at baseline
tp1 → measure at final time point
time_difference (tp0, tp1) → the time elapsed (in years) between the scans tp0 and tp1

To investigate the differences in different neuropsychological test sores among the three groups, Kruskal–Wallis non-parametric analysis of variance (ANOVA) was performed, as the sample size was small.

To investigate the changes in cortical thickness and gray matter volume, we performed two different types of statistical analysis, namely, two-stage regions of interest (ROI)-based analysis, and two-stage voxel-based analysis. In the first stage, we reduced the longitudinal data into a single statistic for each subject using SPC. In the second stage, we compared that single statistic across different groups. To observe any significant changes in 31 predefined ROIs in ROI-based analysis between the meditation and the non-meditation groups, a non-parametric independent sample *t*-test (Mann–Whitney *U*-test) was performed in JASP version 0.14.1 (JASP Team, 2020).

For hippocampal subfields and other subcortical structures such as the cerebellum, thalamus, caudate, putamen, pallidum, and amygdala, SPC in gray matter volume values was estimated for each region. For hippocampal subfields analysis, we normalized each subfield volume with its estimated total intracranial volume (eTIV).


$$V\,o\,l\,u\,m\,e\,{\left(n\,o\,r\,m\,a\,l\,i\,z\,e\,d\right)}_{i}=\,V\,o\,l\,u\,m\,e\,{\left(o\,b\,s\,e\,r\,v\,e\,d\right)}_{i}*\frac{T\,I\,V_{m}}{T\,I\,V_{i}};\left(\text{Du et al., 2007}\right).$$

Where, TIV_m_ → mean total intracranial volume for all subjects
TIV_i_ → total intracranial volume of the ith subject

An independent sample non-parametric *t*-test (Mann–Whitney *U*-test) was used to assess the significant gray matter volume changes between the meditation and non-meditation groups for subcortical structures including all the hippocampal subfields.

Voxel-based statistical analysis for imaging data was performed using Freesurfer’s built-in general linear model (GLM) tool -- Query Design Estimate Contrast (QDEC)^1^. To investigate changes in the cortical thickness and gray matter volume, a longitudinal two-stage voxel-wise whole-brain GLM for both hemispheres was applied. The GLM model compared the SPC values in cortical thickness and gray matter volume between meditation and non-meditation groups. In the GLM model, age, gender, and education were treated as nuisance variables and correction for multiple comparisons was performed in all the analyses. We used the following criterion for considering a region to be significantly changed over time: the cluster should have a corrected false discovery rate (FDR) (Benjamini and Hochberg, 1995) value of *p* < 0.05 and the total size of all the clusters for a given ROI should be more than 50 mm^2^. We calculated the effect size for each ROI which showed any significant change between meditation and non-meditation groups. To compare the between-group differences for all measures, we used the non-parametric Mann–Whitney *U*-test. Rank biserial correlation effect size with a 95% confidence interval was calculated.

## Results

### Neuropsychological Data

Table 1 shows the demographics and baseline global CDR scores of the 32 subjects who completed their second neuropsychological assessment. Baseline scores of age, gender, education, and global CDR scores did not differ significantly between the three groups.

**TABLE 1:** Demographics of participants who completed their neuropsychological assessments at baselines and follow-up.

Variable	Meditation group (*N* = 13)	Non-meditation-focused task group (*N* = 9)	Treatment group (*N* = 10)	Kruskal–wallis test
	
	Median (Q1, Q3)			H- Statistic (*P*-value)
Age at baseline (Years)	67.00(63,68)	68.00(64,75)	68.00(65.5,71.75)	1.723 (0.423)
Education in Years	15.00(14,16)	12.00(12,16)	14.00(10,15.75)	1.541 (0.463)
CDR-Global at baseline	0.5(0.5,0.5)	0.5(0.5,1.0)	0.5(0.5,0.5)	0.477 (0.788)
				Chi-squared test x^2^ (*p*-value)
Gender	F=5(38.46%)	F=6(66.66%)	F=2(20%)	4.319 (0.115)

*Gender: “F” represents the number of female subjects in the respective groups.*

None of the neuropsychological test scores differed significantly between the three groups (Table 2).

**TABLE 2:** Neuropsychological scores (median value) from baseline to follow-up and “*Median (Q1, Q3)*,” “*H-statistic*,” *and* “*P-value*”.

Tests	Meditation group (*N* = 13)	Non-meditation-focused task group (*N* = 9)	Treatment-as-usual (TAU) group (*N* = 10)	Kruskal–wallis test
	
	Median baseline score → median follow-up score: median SPC (Q1, Q3)			H- Statistic (*P*-value)
CDR-Sum of Boxes	2 → 2 −4.101 (−5.917, 6.452)	2.5 → 2 0.00 (−5.825, 3.106)	1.75 → 2.25 0.00 (−5.561, 8.782)	0.166 (0.920)
MOCA	18.5 → 17 −0.677 (−2.277, 0.606)	18 → 18 1.713 (0.5, 2.708)	18 → 21 0.000 (−1.839, 1.009)	3.218 (0.200)
Digit span forward task	5 → 6 0.00 (0.00, 3.190)	4 → 5 0.00 (0.00, 2.347)	5 → 4 −2.946 (−5.97, 0.00)	5.757 (0.056)
Digit span backward task	3 → 4 0.00 (0.00, 0.00)	4 → 4 0.00 (0.00, 0.00)	3.5 → 4 0.00 (0.00, 1.374)	0.244 (0.885)
TMT-A	88 → 99 0.375 (−1.893, 4.593)	115 → 103 −1.348 (−4.69, 0.245)	93 → 100.5 0.233 (−0.127, 1.353)	2.750 (0.253)
TMT-B	300 → 290 0.00 (−0.334, 0.00)	300 → 300 0.00 (0.00, 0.355)	286.5 → 300 0.00 (0.00, 0.914)	0.262 (0.877)
TMT-(B-A)	171 → 148 −0.999 (−3.687, 0.410)	154 → 183 3.820 (−0.857, 4.698)	154 → 121 0.00 (−3.189, 1.423)	3.795 (0.150)
CERAD IR	16 → 19 0.592 (−1.21, 1.77)	19 → 18 1.056 (−0.92, 2.61)	15 → 17.5 2.663 (0.38, 3.61)	2.883 (0.237)
CERAD DR	3 → 4 3.128 (0.00, 5.69)	1 → 3 0.000 (−20.61, 14.56)	4 → 3.5 0.485 (−2.98, 4.00)	0.474 (0.789)
CERAD RECOG	16 → 18 1.109 (0.00, 2.769)	15 → 17 0.000 (−0.925, 2.721)	16.5 → 18 1.674 (−0.317, 3.718)	1.366 (0.505)

*MOCA, Montreal Cognitive Assessment; TMT, Trail Making Task; CERAD, Consortium to Establish a Registry for Alzheimer’s Disease; CERAD (IR), (DR), (RECOG), Immediate Recall, Delayed Recall, Recognition.*

For the 22 subjects used in MRI analysis, baseline scores of age, gender, education, and global CDR scores did not differ significantly between meditation and non-meditation groups (Table 3). The results which meet the statistical criteria are discussed in this manuscript.

**TABLE 3:** Demographics of participants who finished their baseline and follow-up neuroimaging scans.

Variable	Meditation group (*N* = 11)	Non-meditation group (Focused task+treatment) (*N* = 11)	Mann–Whitney *U* test
		
	Median (Q1, Q3)		W (*P*-value)
Age at baseline (Years)	67.00 (64, 68)	71.00 (59.5, 72.5)	41.5 (0.223)
Education in Years	15.00 (13, 16)	14.00 (12, 15.5)	71.5 (0.484)
CDR-Global at baseline	0.50 (0.5, 0.75)	0.50 (0.5, 0.5)	66.0 (0.651)
			Chi-squared test x^2^ (*p*-value)
Gender	F = 4 (36.36%)	F = 5 (45.45%)	0.786 (0.375)

*Gender: “F” represents the number of female subjects in the respective groups.*

### Analysis of Structural Magnetic Resonance Images Data

We compared cortical thickness and gray matter volume between meditation and non-meditation groups at baseline. We did not observe any significant differences between the groups for cortical thickness. We also did not find significant differences in gray matter volume, except in the left lateral occipital, right pars triangularis, and postcentral regions (Please refer to Supplementary Tables 1, 2).

Section “Regions of Interest-Based Analysis” and “Voxel-Wise Analysis” discuss the results of ROI-based and voxel-wise analysis, respectively.

#### Regions of Interest-Based Analysis

We did a comparative ROI-based analysis of cortical thickness and gray matter volume between meditation and non-meditation groups. Results highlighting regions showing significant changes between the meditation and non-meditation groups are shown in Table 4. In contrast with the non-meditation group, the meditation group showed significantly higher cortical thickness and gray matter volume in the left caudal and rostral middle frontal areas. The meditation group showed a significantly higher gray matter volume in left lateral occipital, right inferior parietal, and right superior frontal cortices. On the other hand, we also observed a significant decrease in cortical thickness and gray matter volume mainly in the entorhinal cortex and posterior parts of the brain.

**TABLE 4:** Cortical thickness and gray matter volume changes in ROI-based analysis between the meditation and non-meditation groups in both hemispheres.

ROI name	Meditation group (*N* = 11)	Non-meditation group (Focused task+treatment) (*N* = 11)	Mann–Whitney *U* test
		
	Median SPC (Q1, Q3)		W (*P*-value, r- value)
**Cortical thickness (Meditation > Non-meditation)**			
Left caudal middle frontal	5.517(−0.006,13.246)	−1.606(−15.83,0.37)	96(0.019,0.58)
Left rostral middle frontal	5.23(1.32,17.97)	0.942(−5.93,3.95)	93(0.034,0.53)
**Cortical thickness (Meditation < Non-meditation)**			
Left entorhinal cortex	−9.80(−15.40,−3.54)	−8.46(−3.48,15.36)	27(0.028,−0.55)
Left parahippocampal	−8.46(−18.62,−2.46)	3.33(−7.97,8.11)	28(0.034,−0.53)
Right isthmus cingulate	−12.34(−20.73,−3.05)	−2.00(−4.80,7.98)	30(0.047,−0.50)
Right pericalcarine	−8.48(−11.20,−6.61)	−0.85(−6.05,6.60)	28(0.034,−0.53)
Right posterior cingulate cortex	−11.82(−18.97,2.93)	11.49(0.56,15.20)	29(0.040,−0.52)
**Gray Matter Volume (Meditation > Non-meditation)**			
Left caudal middle frontal	4.47(−2.94,9.98)	−6.38(−29.35,0.080)	95(0.023,0.57)
Left lateral occipital	1.59(−7.93,5.66)	−15.94(−24.76,−5.17)	97(0.016,0.60)
Left rostral middle frontal	6.25(4.08,13.80)	−1.21(−12.63,3.54)	96(0.022,0.58)
Right inferior parietal	0.54(−4.73,4.44)	−8.73(−11.52,−2.99)	93(0.034,0.53)
Right superior frontal	4.98(−1.19,5.28)	−8.84(−15.03,2.12)	91(0.047,0.50)
**Gray Matter Volume (Meditation < Non-meditation)**			
Right entorhinal cortex	−21.29(−38.83,−7.37)	2.29(−13.87,19.97)	27(0.028,−0.55)

*The level of significance (*p-value*), the rank biserial correlation effect size (*r-value*) of the Mann–Whitney *U* test is reported.*

#### Voxel-Wise Analysis

**FIGURE 1:**
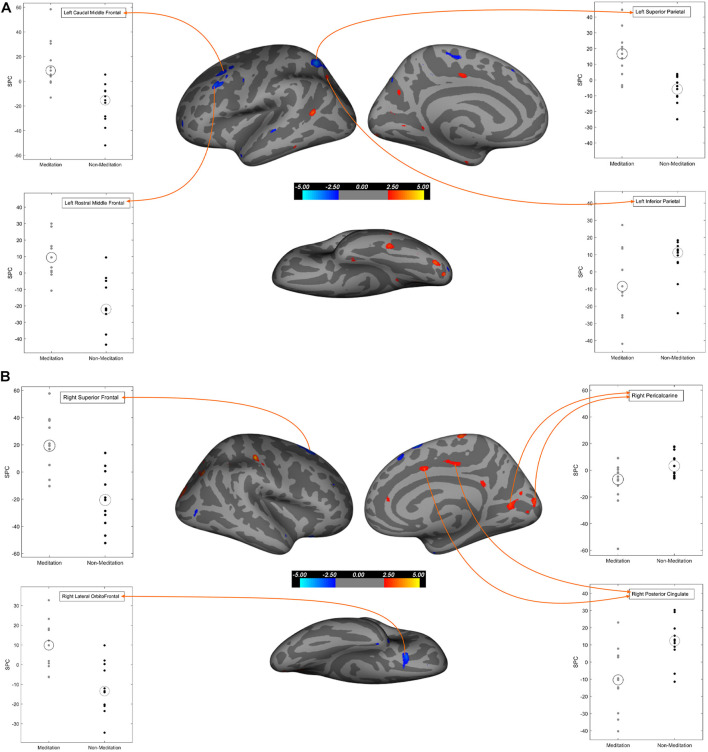
Regions of cortical thickness that exhibit significant differences between meditation and non-meditation cohorts corrected for multiple comparisons, age, gender, and education. In all three different views (Lateral, medial, inferior) of brain surfaces, the blue clusters represent significantly higher SPC values while the red clusters represent significantly lesser SPC values in the meditation group compared to the non-meditation group. Graphs for significantly large cluster sizes are annotated on the inflated brain surface (see Table 5 for more details). **(A)** Left hemisphere; **(B)** Right hemisphere.

**FIGURE 2:**
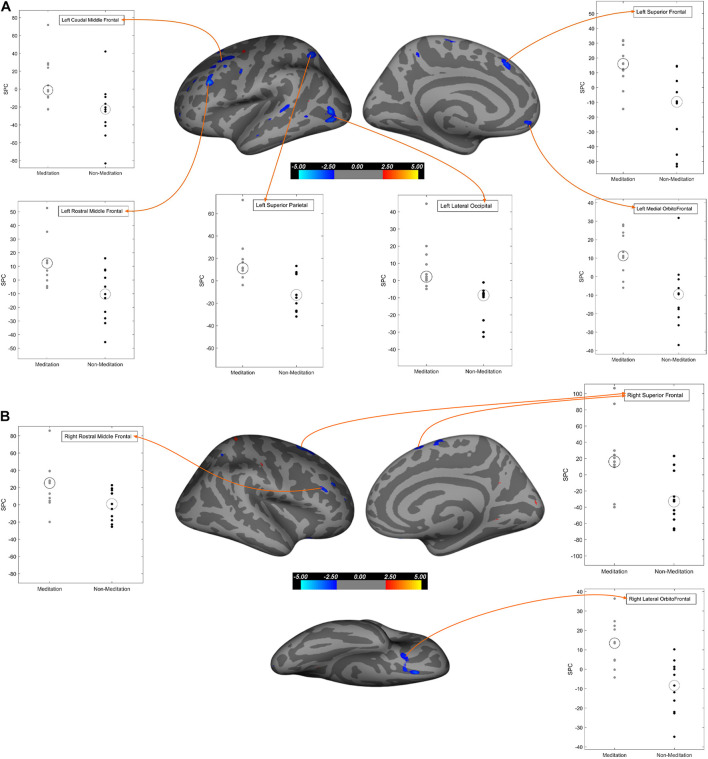
Regions of gray matter volume that exhibit significant differences between meditation and non-meditation cohorts corrected for multiple comparisons, age, gender, and education. In all three different views (Lateral, medial, inferior) of brain surfaces, the blue clusters represent significantly higher SPC values while the red clusters represent significantly lesser SPC values in the meditation group compared to the non-meditation group. Graphs for significantly large cluster sizes are annotated on the inflated brain surface (see Table 6 for more details). **(A)** Left hemisphere; **(B)** Right hemisphere.

To further explore our data, we performed voxel-wise cortical thickness analysis (cortical thickness computed as the shortest distance between the gray/white matter junction and the pial surface), followed by a voxel-wise gray matter volume analysis. Table 5 and Figures 1A,B show the results of the cortical thickness analysis. Table 6 and Figures 2A,B show the results of the gray matter volume analysis.

**TABLE 5:** Brain regions showing significant cortical thickness SPC changes (increase/decrease) in voxel-based analysis between the meditation and non-meditation groups corrected for multiple comparisons, age, gender, and education in both hemispheres.

Brain region (TalX, TalY, TalZ)	Size (mm^2^)	*P*-value

Left Hemisphere		
**SPC for Meditation > Non-meditation**		
Superior parietal (−24.1, −58.9, 53.9)	271.35	<0.00016
Rostral middle frontal (−44.4, 25, 35.6)	128.13	<0.00079
Caudal middle frontal (−33.4, 25, 35.6)	127.14	<0.00157
Paracentral (−8.8, −22, 55.7)	55.79	<0.0047
Caudal middle frontal (−34.1, 7.2, 55.6)	53.52	<0.0030
**SPC for Meditation < Non-meditation**		
Lateral occipital (−30, −83.6, −14.4)	76.78	<0.00041
Inferior parietal (−46.7, −58.4, 10.4)	73.47	<0.00053
Inferior temporal (−45.5, −39.4, 22)	52.6	<0.00153
Superior temporal (−54.8, −23.7, −3.5)	52.19	<0.00226

**Right Hemisphere**		

**SPC for Meditation > Non-meditation**		
Superior frontal (11.7, 13.8, 61.8)	414.92	<0.000067
Lateral orbitofrontal (23, 20.3, −18.5)	123.81	<0.00029
Superior frontal (7.3, 27.9, 51.8)	51.91	<0.0026
**SPC for Meditation < Non-meditation**		
Lateral occipital (24.7, −88, 17.9)	155.11	<0.00054
Pericalcarine (21.8, −71, 7.3)	132.54	<0.00172
Postcentral (48.4, −23.4, 53.7)	115.73	<0.000028
Pericalcarine (6.1, −87.2, 7.5)	104.21	<0.00141
Inferior parietal (33.8, −71.2, 35.5)	84.82	<0.000035
Paracentral (4.6, −26.3, 68)	69.77	<0.00046
Posterior cingulate (13.4, −20, 38)	56.88	<0.0047

*TalX, Y, and Z represent Talairach coordinates X, Y, and Z. Table includes only the regions which had cluster size greater than 50 mm^2^ and corresponding significance (*p-value*) less than 0.05.*

**TABLE 6:** Brain regions showing significant gray matter volume SPC changes (increase/decrease) in voxel-based analysis between the meditation and non-meditation groups corrected for multiple comparisons, age, gender, and education in both hemispheres.

Brain region (TalX, TalY, TalZ)	Size (mm^2^)	*P*-value

Left Hemisphere		
**SPC for Meditation > Non-meditation**		
Lateral occipital (−42.6, −75.9, 4.5)	281.47	<0.00021
Caudal middle frontal (−32.4, 13.3, 47.6)	225.47	<0.0025
Rostral middle frontal (−44.1, 26.9, 28.1)	136.1	<0.000073
Superior parietal (−25.2, −57.3, 51.6)	127.19	<0.0010
Superior frontal (−6.9, 26.5, 45.2)	90.77	<0.0013
Banks of superior temporal sulcus (−62, −34.3, 4.6)	87.22	<0.0027
Medial orbitofrontal (−9.1, 47.8, −8.8)	64.64	<0.0020
Superior parietal (−15.1, −68.2, 47.2)	53.04	<0.0011
Caudal middle frontal (−32.2, 23.8, 42)	48.32	<0.0040

**Right Hemisphere**		

**SPC for Meditation > Non-meditation**		
Superior frontal (18.6, 14, 60.2)	341.4	<0.0002
Lateral orbitofrontal (26.7, 18.1, −20.5)	70.23	<0.0027
Superior frontal (10, −3.7, 67)	66.56	<0.0030
Rostral middle frontal (44.7, 31.8, 21.6)	62.13	<0.0014
Lateral orbitofrontal (13.2, 31.7, −17.8)	50.7	<0.0005

*TalX, Y, and Z represent Talairach coordinates X, Y, and Z. Table includes only the regions which had cluster size greater than 50 mm^2^ and corresponding significance (*p-value*) less than 0.05. None of the brain regions showed significantly decreased gray matter volume in the meditation group. The medial orbitofrontal region showed negative SPC values of gray matter volume for both groups but significantly less negative in the meditation group compared to the non-meditation group.*

**FIGURE 3:**
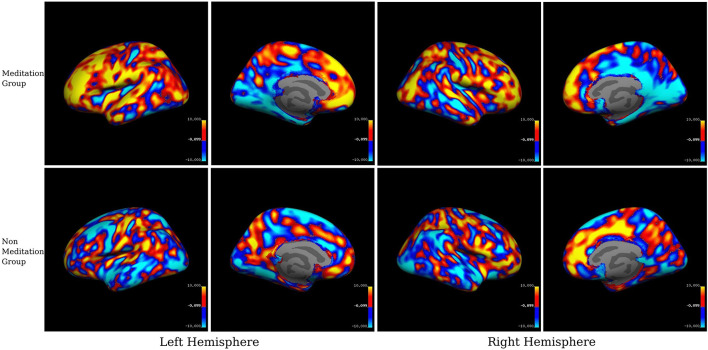
Within-group averaged SPC changes for cortical thickness in the meditation (Group1) and the non-meditation (Groups2+3) groups. Positive values (red-yellow) correspond to the increased cortical thickness and negative values (light blue-dark blue) corresponds to decreased cortical thickness. The voxels with SPC between –0.1 and +0.1 have been left uncolored. The top panel represents the meditation group while the lower panel represents the non-meditation group.

**FIGURE 4:**
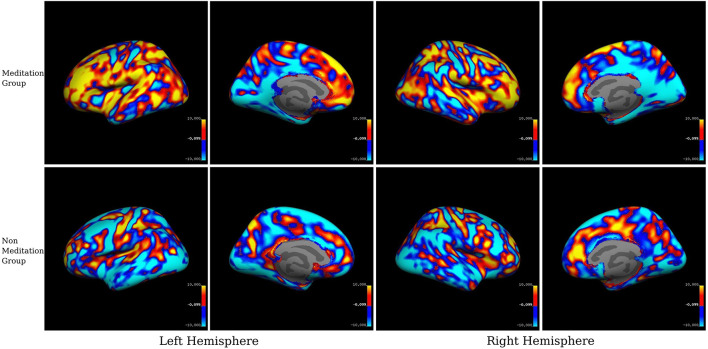
Within-group averaged SPC changes for gray matter volume in the meditation (Group1) and the non-meditation (Groups2+3) groups. Positive values (red-yellow) corresponds to the increased gray matter volume and negative values (light blue-dark blue) corresponds to decreased gray matter volume. The voxels with SPC between –0.1 and +0.1 have been left uncolored. The top panel represents the meditation group while the lower panel represents the non-meditation group.

For the visual inspection of increased or decreased cortical thickness and gray matter volume within each group, we displayed the mean SPC values in each group on inflated brain surfaces (see Figures 3, 4).

##### Cortical Thickness

Our results (Table 5 and Figures 1A,B, 3) showed significant effects of meditation mostly in the anterior parts of the brain, selectively in the frontal regions and in the occipitoparietal region in the posterior part. The meditation group showed significantly increased cortical thickness in the left superior parietal, left caudal middle frontal, left rostral middle frontal, left paracentral and in the right hemisphere, superior frontal, and lateral orbitofrontal regions. The meditation group also showed decreased thickness compared with the non-meditation group in the left lateral occipital, inferior parietal, inferior temporal, and superior temporal cortices, and on the right hemisphere in the lateral occipital, pericalcarine, postcentral, inferior parietal, paracentral, and posterior cingulate cortices. Changes in cortical thickness were observed asymmetrically over multiple regions either in the right or the left hemisphere, except in the inferior parietal and lateral occipital cortices where they were seen bilaterally.

##### Gray Matter Volume

In the whole-brain gray matter volume analysis (Table 6 and Figures 2A,B, 4), in contrast to the non-meditation group, the meditation group showed significantly higher gray matter volume bilaterally in the rostral middle frontal and superior frontal cortices. In the left hemisphere, the superior parietal and caudal middle frontal regions showed an increase in volume, as did the lateral orbitofrontal region in the right hemisphere. As shown in Figure 2A, the left superior frontal changes were observed mainly in the dorsomedial frontal regions. None of the brain regions showed significantly decreased gray matter volume in the meditation group compared to the non-meditation group.

#### Volume Analysis of the Subcortical Structures

On comparing the volume of subcortical structures including the cerebellum, thalamus, caudate, putamen, pallidum, and amygdala between the meditation and the non-meditation group, no significant changes in the left hemisphere were observed. However, we observed significantly increased volume in the right thalamus (*p* < 0.034) and a non-significant increasing trend in the right caudate (*p* < 0.056) in the meditation group.

##### Hippocampal Subfields Analysis

**FIGURE 5:**
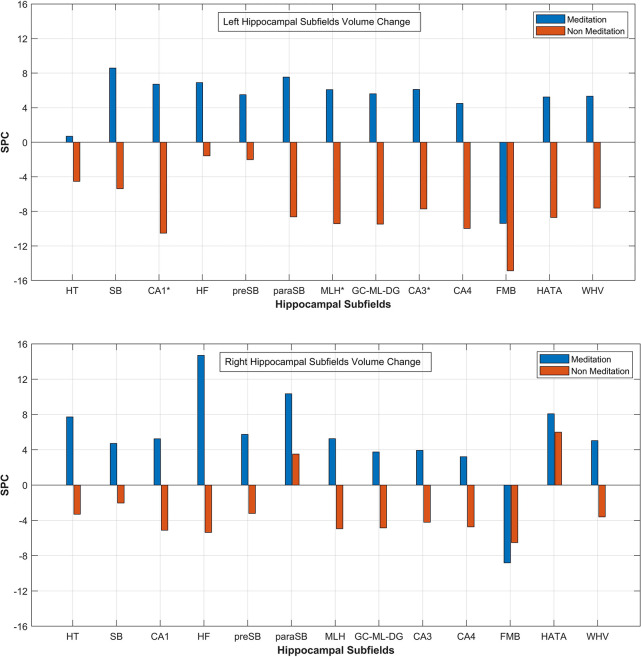
Mean SPC values for volume of each Left and Right Hippocampal Subfields respectively for the mediation and the non-meditation groups. HT, hippocampal tail; SB, subiculum; CA1, HF, hippocampal fissure; preSB, presubiculum; paraSB, parasubiculum; MLH, molecular layer HP; GC-ML-DG, [Granule Cell (GC) and Molecular Layer (ML) of the Dentate Gyrus (DG)]; FMB, fimbria; HATA (hippocampus-amygdala-transition-area); WHV, Whole Hippocampal Volume. *Corresponds to significant changes in the subfield’s volume.

From the statistical analysis, we observed that the total hippocampal volume showed a positive rate of change in both the hemispheres from base to repeat scans in the meditation group, while in the non-meditation group, it decreased. Selectively, the volume of individual subfields in both hemispheres showed a positive change in the mediation group compared to the non-mediation group except in the fimbria (Figure 5). The left CA1 (*p* < 0.001), left molecular layer HP (*p* < 0.04), and left CA3 (*p* < 0.040) showed significantly higher SPC volume in the meditation group compared to the non-meditation group. The mean SPC values of each hippocampal subfield for the meditation and the non-meditation groups for both the hemispheres are plotted in Figure 5.

## Discussion

In this longitudinal study, we explored the effect of long-term daily meditation on the structural and neuropsychological changes in patients with MCI and mild AD. Our results show significant morphometric changes in cortical gray matter in the meditation group in a pattern that provides support to our hypotheses.

We used both ROI-based and voxel-wise analyses for our study. In *within-group* analysis between two-time points, we found a widespread increase in cortical thickness or gray matter volume in the meditation group, particularly prominently over the left prefrontal cortex, but also on the right. Conversely, we found reduced cortical thickness and gray matter volume following meditation in the posterior cingulate cortex, occipital cortex, and medial temporal regions, especially in the right hemisphere. *Between-group* comparison of meditators and non-meditators using ROI data revealed that meditation significantly increased cortical thickness and gray matter volume in the caudal and rostral middle frontal cortices bilaterally and increased gray matter volume alone over the right superior frontal gyrus (SFG). Significantly reduced cortical thickness, gray matter volume, or both was seen over bilateral entorhinal and left parahippocampal cortices, right PCC and isthmus of cingulate, and right pericalcarine cortex.

A subsequent voxel-wise comparison over the prefrontal cortex showed significantly higher cortical thickness or gray matter volume in the left caudal middle frontal, bilateral rostral middle frontal, bilateral superior frontal, right lateral orbitofrontal, and left medial orbitofrontal cortices in the meditation group. The caudal middle frontal cortex is important for top-down modulation of attention such as in cognitive decision-making when confronted with competing visual stimuli or for re-orienting attention from stimulus-driven exogenous control to goal-directed endogenous control (Japee et al., 2015; Germann and Petrides, 2020). Cortical thinning in the caudal middle frontal region has been associated with poorer scores on attention and divided attention (Grambaite et al., 2011). It is therefore interesting that sustained meditation practice could strengthen the caudal middle frontal cortices in our patients. The rostral middle frontal cortex is important for attention, working memory, executive control, and emotion regulation (Miller and Cohen, 2001; Michalski et al., 2017) and are activated during response inhibition and switching performances (Grambaite et al., 2011). We speculate that the significantly increased cortical thickness and gray matter volume in the rostral middle frontal cortices in our patients may have followed repeated reinforcement of many of these functions by long-term meditation.

Previous works on healthy meditators have shown an increased cortical thickness or gray matter volume in the ventromedial orbitofrontal prefrontal cortex (Kang et al., 2013; Hernández et al., 2016). Unlike in healthy subjects, the orbitofrontal cortex might be thinner in patients with MCI and AD (Zhao et al., 2015). Our finding of a reversal of thinning of the orbitofrontal cortex in meditators is encouraging and suggests that long-term practice of meditation could reinforce the regions that control social cognition and behavior (Campos et al., 2019). The lateral orbitofrontal cortex has been associated with the evaluation and prediction of possible outcomes of an action. The medial orbitofrontal cortex takes this information and compares and contrasts the choices based on value, before selecting the appropriate action, possibly in conjunction with the dorsolateral prefrontal cortex (Rudebeck and Murray, 2014). One of the distinctive features of meditation on breath or on witnessing thoughts is the default actions of mind-wandering or rise and fall of thoughts, respectively. The mind repeatedly gravitates out of attention and requires constant updating, the evaluation of choices, and value-based selection to bring it back to the task at hand. Although we are not aware of specific physiological studies on the role of the orbitofrontal cortex in meditation, it is conceivable how meditation could potentially help reinforce the functions of the lateral and medial orbitofrontal cortices.

The superior frontal gyrus (SFG) has been associated with working memory (du Boisgueheneuc et al., 2006; Nissim et al., 2017), cognitive control, cognitive execution, and motor control (Li et al., 2013) and spatial cognition orientation (du Boisgueheneuc et al., 2006). Lack of cognitive control by the SFG has been implicated in impulsivity (Hu et al., 2016) and poor social functioning (Tully et al., 2014). The increased cortical thickness or gray matter volume in our subjects could be because of the sustained control achieved by meditation over externally or internally arising distractions.

The meditation group showed significantly increased cortical thickness of the left superior parietal lobule and decreased cortical thickness of bilateral inferior parietal lobules. The superior parietal lobule, the intraparietal sulcus, and the frontal eye field form parts of the dorsal attentional network that controls top-down, goal-oriented visual attention (Corbetta and Shulman, 1998; Kastner et al., 1999). The right inferior parietal lobule (including the temporoparietal junction) is part of the ventral attentional network that mediates bottom-up responses driven by salient or unexpected stimuli (Corbetta et al., 2008). The two networks appear to be controlled by a “circuit breaker,” allowing the required flexibility and balance between attending to the goal-oriented responses and stimulus-driven, potentially distracting responses (Corbetta and Shulman, 2002; Behrmann et al., 2004; Fox et al., 2006; Shomstein, 2012; Japee et al., 2015). Meditation, therefore, appears to have strengthened regions subserving top-down control, while attenuating those that oversee stimulus-driven responses.

Significantly more thinning of the right PCC in our meditation group parallels similar findings by others, albeit using a different meditation technique (Kang et al., 2013). The PCC is central to the functioning of the DMN and is active during mind wandering, daydreaming, planning for the future, and retrieval of autobiographical memory. Modulation of the PCC activity is important for effectively switching from a broad, unrestrained attention to the more sharply focused attention to a salient external stimulus (Sridharan et al., 2008; Menon and Uddin, 2010; Leech and Sharp, 2014). The PCC and the hippocampus lose structural and functional integrity early in the course of AD (Greicius et al., 2004) thereby not only affecting autobiographical and episodic memory but also weakening the anticorrelation of the DMN with the frontoparietal attentional networks. In addition to the right PCC, significantly more cortical thinning or shrinkage of gray matter volume in meditators was also seen in the left parahippocampal cortex and the entorhinal cortices bilaterally, along with similar changes in the inferior parietal cortices, mentioned earlier. It is conceivable that meditation, by enhancing attentional and executive control and by reducing mind wandering, further shifts neural activity away from the already degenerated posterior DMN structures to the prefrontal cortex. However, the hippocampal substructures themselves tended to show a greater increase in volume in the meditation group, and significant increases were seen in the left CA1, CA3, and the molecular layer when compared with the non-meditation group. This seems counterintuitive and difficult to explain easily with our current data. Nevertheless, the effect of meditation in slowing down hippocampal volume loss in MCI patients has been described earlier (Wells et al., 2013b).

Our finding of lesser cortical thickness in bilateral lateral occipital cortices, right pericalcarine cortex, as well as the post-central cortex in the meditating group is consistent with the anterior-posterior gradient seen in meditation, with more cortical thinning in posterior brain regions (Kang et al., 2013). Given that occipital thinning in these regions has been reported in MCI and AD (Long et al., 2018; Yang et al., 2019; Edmonds et al., 2020), it is possible that our meditation techniques, by reinforcing the anterior predominant regions responsible for top-down control and suppressing the posterior bottom-up stimulus-driven regions, further promoted the most optimal use of limited cognitive resources.

It would be interesting to consider whether the relatively less thinning in the posterior brain regions in non-meditators could have partly resulted from the positive effect on these structures in the coloring task group. Indeed, the right posterior cingulate cortex was thicker in non-meditators at follow-up. Although we are not aware of previous morphometric studies in this context, functional connectivity studies have shown increased activation of the PCC/precuneus and its frontal and parietal connections following visual art production (Bolwerk et al., 2014). However, the subjects engaged in our coloring task were too few and any further speculation can only be done after a larger study. Moreover, even though both meditation and the coloring tasks require attention and top-down control, meditation, by actively discouraging mind wandering, may lead to increased suppression of the posterior brain regions and consequent loss of gray matter, as seen in our study and by others (Kang et al., 2013).

Among the subcortical structures, only the right thalamus showed a significant volume increase among the meditators. This is in keeping with previous reports on the positive effects of meditation on the thalamus (Luders et al., 2009; Newberg et al., 2010; Laneri et al., 2016). A possible explanation for this is the heightened sensory awareness during meditation (Laneri et al., 2016). This allows the meditator to observe a continual flow of sensory stimuli that is a part of the meditation experience.

Contrary to the positive structural changes in the brain, we could not demonstrate significant cognitive improvement following meditation. This is despite increases in gray matter in areas known to control attention, executive function, and memory. Nevertheless, we observed a trend toward improved attention in meditators in the digit forward task. In a recent study (Yu et al., 2021), significantly improved attention, and better performance in color trails interference task were seen after 9 months of mindfulness meditation in MCI subjects, but the effects were not seen after correcting for false discovery rate. No other neuropsychological function changed significantly in that study during this period (Yu et al., 2021). Indeed, results from the few studies that have looked at the cognitive effects of meditation in MCI or AD patients have been variable, with some showing no significant improvement compared to controls (Wells et al., 2013a; Larouche et al., 2018, 2019; Klainin-Yobas et al., 2019). It is possible that a longer follow-up would bring out the cognitive benefits, as has been noted in some of the studies (Quintana-Hernández et al., 2016; Wong et al., 2017). Alternatively, the standard neuropsychological tests being used may not have been appropriate to pick up early cognitive changes in these subjects.

To ensure robust and meaningful morphometric analysis with a limited sample size, we chose to report only those regions showing significant difference between the meditation and non-meditation groups and having a minimum cluster size of over 50 mm^2^. The large effect sizes of more than 0.5 in all such regions in the ROI based analysis is comparable to the effect sizes reported by others (Yang et al., 2016; Yu et al., 2021).

### Limitations of the Study

The high attrition due to the pandemic in our study meant that we had to work with a smaller sample than planned and had to club the control groups together for our analysis. It is possible that our results could have been different with a bigger sample. Although we cannot rule out some attrition bias contributing to our results, the dropouts were across the groups, the majority occurred early in the study, and the later ones were uniformly out of fear of the pandemic. All dropouts, either from neuropsychology tests or imaging, were excluded from the respective data analysis. Also, the final sample retained the originally planned ratio among the groups.

We recognize that an objective measure of the degree of training achieved by each participant would have been useful. However, our training sessions were on a one-to-one basis, and at the end of each training session, we did ask the subject to discuss his/her performance for the day, and every effort was made by the trainer to clear all doubts. A home-based longitudinal study must rely on self-reporting and carries a reporting bias. Our patients were encouraged to keep a log of their practice, but few maintained this regularly. However, we maintained regular telephonic contact with the patients in the meditation group and the active control group, and with their caregivers, to check for compliance and to address queries related to the task. In addition, patients in the active control group were required to submit their coloring books before collecting the next one.

For the hippocampal subfields analysis, we have used isotropic 1 mm^3^ T1-w images using automatic segmentation of Freesurfer 6.0. It is possible that higher resolution images could have provided more accurate results (Wisse et al., 2021).

We also acknowledge that the results of a non-randomized study with consecutive sampling are subject to selection bias. Our observations will need to be confirmed by future large randomized-control studies designed to address these questions.

## Conclusion

In this study, we investigated the effects of long-term meditation on structural brain imaging parameters of MCI and mild AD patients using surface-based morphometric analysis. An increase in gray matter predominantly over the prefrontal cortex and a decrease more posteriorly suggest a shift toward increasing top-down control in the meditators. More importantly, it suggests that long-term daily meditation may be able to slow-down the neurodegenerative process in crucial areas of the brain. The lack of objective benefit in neuropsychological tests is not necessarily disheartening and may become evident after longer periods of observation, or earlier by using person-centered tests that can evaluate cognition in day-to-day tasks. Unlike previous studies on the subject, we employed a silent, sitting meditation technique that excluded mantras, yogic exercises, or body movements. We could therefore include a longer meditation routine than those used by some other researchers. To our knowledge, this is also the first study on morphometric changes caused by meditation to include patients with AD. Our results need to be validated on a larger cohort observed at more time points. Investigation of other imaging modalities to analyze structural changes, especially the fiber pathways connecting different regions as well as their relation to the functional connectivity changes would offer us a more comprehensive account of the longitudinal effects of meditation intervention in neurodegenerative diseases.

## Data Availability Statement

The original contributions presented in the study are included in the article/Supplementary Material, further inquiries can be directed to the corresponding author/s.

## Ethics Statement

The studies involving human participants were reviewed and approved by Ethics Committee, Apollo Gleneagles Hospital, Kolkata, India. The patients/participants provided their written informed consent to participate in this study.

## Author Contributions

AG and ND conceptualized the study and arranged for funding. AG and RSB supervised the study. AG, ND, JB, RB, and MG contributed to the study design. AG, ND, MeD, and GP acquired the data. MaD, AP, ND, AG, and RSB contributed to data analysis and interpretation. MaD, AP, ND, and AG drafted the manuscript. The manuscript was revised by all authors and approved the submitted version.

## Conflict of Interest

The authors declare that the research was conducted in the absence of any commercial or financial relationships that could be construed as a potential conflict of interest.

## Publisher’s Note

All claims expressed in this article are solely those of the authors and do not necessarily represent those of their affiliated organizations, or those of the publisher, the editors and the reviewers. Any product that may be evaluated in this article, or claim that may be made by its manufacturer, is not guaranteed or endorsed by the publisher.

## Footnotes

1
https://surfer.nmr.mgh.harvard.edu/fswiki/FsTutorial/QdecGroupAnalysis_freeview
