# Supplementary material for: Effects of Meditation on Structural Changes of the Brain in Patients With Mild Cognitive Impairment or Alzheimer’s Disease Dementia
Source: Front Hum Neurosci. 2021 Nov 12;15:728993. doi: 10.3389/fnhum.2021.728993 (PMC8633496; doi:10.3389/fnhum.2021.728993)
Supplement: Supplementary file 1 [file Data_Sheet_1.pdf]

Table 1. Baseline results on comparing the cortical thickness of the meditation group with the non-meditation group. The 'lh' and 'rh' represent the left and right hemispheres respectively. bank ssts: banks of the superior temporal sulcus.

ROIs	P-value	ROIs	P-value
lh_bank ssts	0.949	rh_bank ssts	0.898
lh_caudal anterior cingulate	0.699	rh_caudal anterior cingulate	0.847
lh_caudal middle frontal	0.562	rh_caudal middle frontal	0.562
lh_cuneus	1	rh_cuneus	0.699
lh_entorhinal	0.478	rh_entorhinal	0.652
lh_fusiform	0.365	rh_fusiform	0.699
lh_inferior parietal	0.699	rh_inferior parietal	0.898
lh_inferior temporal	0.797	rh_inferior temporal	0.847
lh_isthmus cingulate	0.193	rh_isthmus cingulate	0.332
lh_lateral occipital	0.27	rh_lateral occipital	0.332
lh_lateral orbitofrontal	0.243	rh_lateral orbitofrontal	0.797
lh_lingual	0.401	rh_lingual	0.478
lh_medial orbitofrontal	0.562	rh_medial orbitofrontal	0.401
lh_middle temporal	0.949	rh_middle temporal	0.699
lh_parahippocampal	0.797	rh_parahippocampal	0.332
lh_paracentral	1	rh_paracentral	0.519
lh_pars opercularis	0.847	rh_pars opercularis	0.332
lh_pars orbitalis	0.365	rh_pars orbitalis	0.606
lh_pars triangularis	0.949	rh_pars triangularis	0.797
lh_pericalcarine	1	rh_pericalcarine	1
lh_postcentral	0.606	rh_postcentral	0.332
lh_posterior cingulate	0.193	rh_posterior cingulate	0.519
lh_precentral	0.898	rh_precentral	0.27
lh_precuneus	0.652	rh_precuneus	0.365
lh_rostral anterior cingulate	0.562	rh_rostral anterior cingulate	0.116

lh_rostral middle frontal	1	rh_rostral middle frontal	0.949
lh_superior frontal	0.748	rh_superior frontal	0.699
lh_superior parietal	0.797	rh_superior parietal	0.898
lh_superior temporal	0.797	rh_superiortemporal	0.898
lh_supramarginal	0.797	rh_supramarginal	0.847
lh_frontal pole	0.748	rh_frontal pole	0.652
lh_temporal pole	0.847	rh_temporal pole	0.797
lh_transverse temporal	0.898	rh_transverse temporal	0.606
lh_insula	0.365	rh_insula	0.478

Table 2. Baseline results on comparing the gray matter volume of the meditation group with the non-meditation group. The 'lh' and 'rh' represent the left and right hemispheres respectively. bank ssts: banks of the superior temporal sulcus.

ROIs	P-value	ROIs	P-value
lh_bank ssts	0.652	rh_bank ssts	0.652
lh_caudal anterior cingulate	0.898	rh_caudal anterior cingulate	0.562
lh_caudal middle frontal	0.217	rh_caudal middle frontal	0.797
lh_cuneus	0.847	rh_cuneus	0.748
lh_entorhinal	0.133	rh_entorhinal	0.898
lh_fusiform	0.606	rh_fusiform	0.652
lh_inferior parietal	0.652	rh_inferior parietal	0.699
lh_inferior temporal	0.699	rh_inferior temporal	0.606
lh_isthmus cingulate	0.217	rh_isthmus cingulate	0.065
**lh_lateral occipital**	**0.034**	rh_lateral occipital	0.27
lh_lateral orbitofrontal	0.699	rh_lateral orbitofrontal	0.519
lh_lingual	1	rh_lingual	0.478
lh_medial orbitofrontal	0.847	rh_medial orbitofrontal	0.365
lh_middle temporal	0.748	rh_middle temporal	0.478
lh_parahippocampal	0.519	rh_parahippocampal	0.606
lh_paracentral	0.243	rh_paracentral	0.076

lh_pars opercularis	1	rh_pars opercularis	0.438
lh_pars orbitalis	1	rh_pars orbitalis	0.949
lh_pars triangularis	0.898	**rh_pars triangularis**	**0.04**
lh_pericalcarine	0.365	rh_pericalcarine	0.438
lh_postcentral	0.562	**rh_postcentral**	**0.028**
lh_posterior cingulate	0.27	rh_posterior cingulate	0.438
lh_precentral	0.898	rh_precentral	0.652
lh_precuneus	0.652	rh_precuneus	1
lh_rostral anterior cingulate	0.562	rh_rostral anterior cingulate	0.438
lh_rostral middle frontal	0.401	rh_rostral middle frontal	0.27
lh_superior frontal	1	rh_superior frontal	0.606
lh_superior parietal	0.133	rh_superior parietal	0.652
lh_superior temporal	0.898	rh_superior temporal	0.949
lh_supramarginal	0.847	rh_supramarginal	1
lh_frontal pole	0.797	rh_frontal pole	0.116
lh_temporal pole	1	rh_temporal pole	0.847
lh_transverse temporal	0.243	rh_transverse temporal	0.898
lh_insula	0.171	rh_insula	0.217
